# Smooth muscle cells differentiated from mesenchymal stem cells are regulated by microRNAs and suitable for vascular tissue grafts

**DOI:** 10.1074/jbc.RA118.001739

**Published:** 2018-04-11

**Authors:** Wenduo Gu, Xuechong Hong, Alexandra Le Bras, Witold N. Nowak, Shirin Issa Bhaloo, Jiacheng Deng, Yao Xie, Yanhua Hu, Xiong Z. Ruan, Qingbo Xu

**Affiliations:** From the ‡ School of Cardiovascular Medicine & Science, King's College London, British Heart Foundation Centre, London SE5 9NU, United Kingdom and; the § Centre for Lipid Research, Key Laboratory of Molecular Biology for Infectious Diseases (Ministry of Education), Department of Infectious Diseases, the Second Affiliated Hospital, Chongqing Medical University, Centre for Nephrology, University College London, Rowland Hill Street, London NW3 2PF, United Kingdom

**Keywords:** mesenchymal stem cells (MSCs), vascular smooth muscle cells, cell differentiation, microRNA mechanism, transforming growth factor beta (TGF-β), tissue engineering, umbilical cord mesenchymal stem cells

## Abstract

Tissue-engineered vascular grafts with long-term patency are greatly needed in the clinical settings, and smooth muscle cells (SMCs) are a critical graft component. Human mesenchymal stem cells (MSCs) are used for generating SMCs, and understanding the underlying regulatory mechanisms of the MSC-to-SMC differentiation process could improve SMC generation in the clinic. Here, we found that in response to stimulation of transforming growth factor-β1 (TGFβ1), human umbilical cord–derived MSCs abundantly express the SMC markers α-smooth muscle actin (αSMA), smooth muscle protein 22 (SM22), calponin, and smooth muscle myosin heavy chain (SMMHC) at both gene and protein levels. Functionally, MSC-derived SMCs displayed contracting capacity *in vitro* and supported vascular structure formation in the Matrigel plug assay *in vivo*. More importantly, SMCs differentiated from human MSCs could migrate into decellularized mouse aorta and give rise to the smooth muscle layer of vascular grafts, indicating the potential of utilizing human MSC-derived SMCs to generate vascular grafts. Of note, microRNA (miR) array analysis and TaqMan microRNA assays identified miR-503 and miR-222-5p as potential regulators of MSC differentiation into SMCs at early time points. Mechanistically, miR-503 promoted SMC differentiation by directly targeting SMAD7, a suppressor of SMAD-related, TGFβ1-mediated signaling pathways. Moreover, miR-503 expression was SMAD4-dependent. SMAD4 was enriched at the miR-503 promoter. Furthermore, miR-222-5p inhibited SMC differentiation by targeting and down-regulating ROCK2 and αSMA. In conclusion, MSC differentiation into SMCs is regulated by miR-503 and miR-222-5p and yields functional SMCs for use in vascular grafts.

## Introduction

Vascular bypass or replacement surgery is an important approach for the treatment of severe vascular diseases (1). Engineering vascular grafts with long-term patency is a promising technique to meet the clinical needs. Several types of stem cells have been explored to generate enough vascular cells for vascular graft construction (2–4). Vascular cells derived from c-kit–positive mouse embryonic stem cells and pluripotent stem cells reprogrammed from fibroblasts have been used to engineer functional small-diameter blood vessels, which upon transplantation *in vivo* demonstrated improved patency and decreased mortality rate compared with grafts without cell seeding (2, 5). However, the primary limitation of using these cells is the potential risk of tumorigenesis and the difficulty in obtaining a sufficient number of SMCs.^4^

Human mesenchymal stem cells (MSCs) have emerged as a promising cell source for vascular tissue engineering because they are multipotent and display immune modulation characteristics. Such features enable the use of MSCs to engineer vascular grafts seeded with allogeneic cells (6). MSCs could be isolated from multiple tissues (7). In addition to multiple differentiation capacities toward adipocytes, osteocytes, and chondrocytes, MSCs are also capable of differentiating toward vascular lineages such as SMCs (8) and endothelial cells (9). Previous efforts of mesenchymal stem cell differentiation toward SMCs have mainly focused on MSCs originating from bone marrow and adipose tissue (10, 11). Our study is the first to explore the SMC differentiation capacity of MSCs from the human umbilical cord. MSCs from umbilical cord present a significant advantage for vascular tissue engineering due to their higher proliferation capacity compared with MSCs from the bone marrow (12). More importantly, from a translational perspective, the umbilical cord could be frozen upon birth and stored for future use later in life (13). Our study also uncovered new mechanisms involved in the SMC differentiation from MSCs, a process still poorly understood to this day.

MicroRNAs (miRNAs) are short noncoding RNAs that participate in regulating multiple important biological processes. Several microRNAs have been identified to be related to SMC differentiation during the development and the maturation of vascular SMC phenotypes among which miR-143/145 are the most extensively studied (14, 15). miR-143/145 are up-regulated upon TGFβ1 treatment, which suggests the interaction of miRNAs and conventional signaling pathways in SMC phenotype switching (16). However, little is known about the involvement of miRNAs in mesenchymal stem cell differentiation to SMCs. Currently, more therapeutic strategies involving miRNAs are paving their way to the clinics (17, 18), and a better understanding of the role of miRNA in SMC differentiation could have implications for vascular graft tissue engineering to treat vascular diseases.

In this study, MSCs from human umbilical cord were utilized to generate functional SMCs for vascular tissue engineering. We demonstrated that these MSC-differentiated cells exhibited typical properties of functional SMCs, including increased contractility and vasculogenesis capacity. Moreover, we showed that functional vascular grafts could be generated utilizing the SMCs differentiated from human umbilical cord MSCs. Finally, miRNA-centered mechanisms involved in the differentiation process into SMCs were elucidated with the identification of novel regulatory miRNAs (miR-503-5p and miR-222-5p).

## Results

### Functional SMCs can be derived from human MSCs upon TGFβ1 stimulation

**Figure 1.**
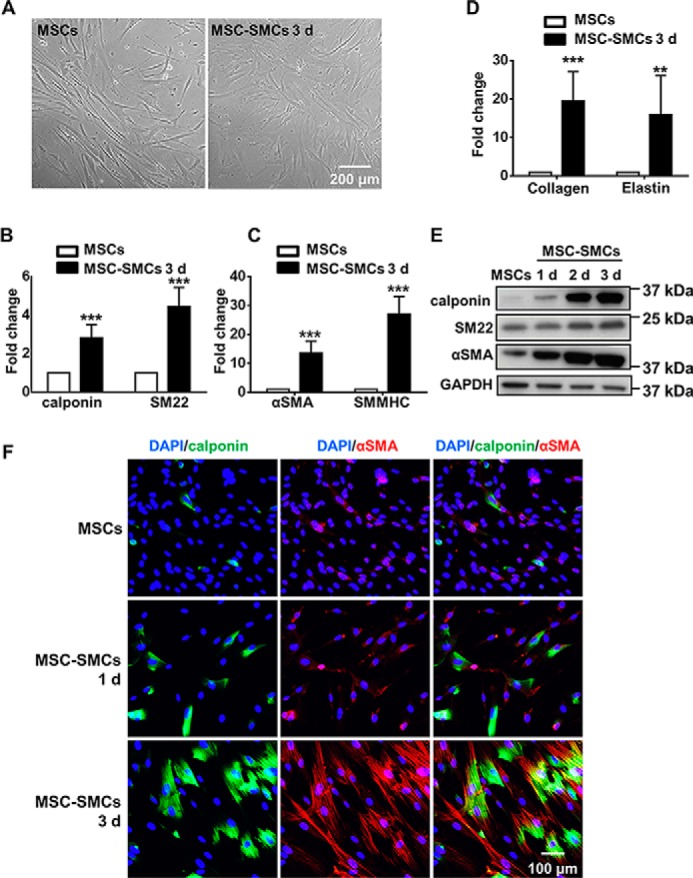
**Differentiation of human umbilical cord MSCs toward SMC lineage with TGFβ1.** *A,* morphology of undifferentiated MSCs and cells cultured in differentiation medium (αMEM with 5 ng/ml TGFβ1 and 1% serum) for 3 days (*d*) (MSC-SMCs 3d). *B–D,* Q-PCR showed the mRNA level induction of calponin, SM22, αSMA, SMMHC, collagen I, and elastin in the differentiation medium for 3 days. *E,* Western blot analysis showed the induction of SMC specific markers at different time points during differentiation at the protein level. Images shown are representative of three independent experiments. *F,* immunofluorescent staining showed the induction of SMC-specific markers at different time points. Representative images are shown from three independent experiments. Data are presented as the mean ± S.D. and are from three independent experiments. **, *p* < 0.01, and ***, *p* < 0.001. *MSC-SMCs*, smooth muscle cells differentiated from mesenchymal stem cells.

To establish the optimal conditions to induce SMC differentiation from human MSCs, the concentration of serum and TGFβ1 were first optimized (data not shown). Treatment of MSCs with 5 ng/ml TGFβ1 in αMEM with 1% serum induced the optimal differentiation toward SMC lineages. MSC-derived SMCs (MSC-SMCs) exhibited SMC-like expanded and elongated spindle-shaped morphology compared with undifferentiated cells (Fig. 1*A*). Typical SMC markers, including calponin, SM22, αSMA, and SMMHC (Fig. 1, *B* and *C*), were up-regulated at the mRNA level in MSCs placed in differentiation medium for 3 days. Furthermore, mRNA of genes related to extracellular matrix synthesis (collagen I and elastin) was also elevated (Fig. 1*D*). Western blot analysis demonstrated the up-regulation of SMC-specific markers (calponin, SM22, and αSMA) in a time-dependent manner at the protein level (Fig. 1*E*). Moreover, immunofluorescent staining confirmed the result of Western blotting and showed the co-localization of calponin and αSMA (Fig. 1*F*). In addition, differentiated SMCs from MSCs showed comparable SMC marker level compared with *in vitro* cultured human aortic SMCs, demonstrating the differentiation efficiency (Fig. S1*A*).

**Figure 2.**
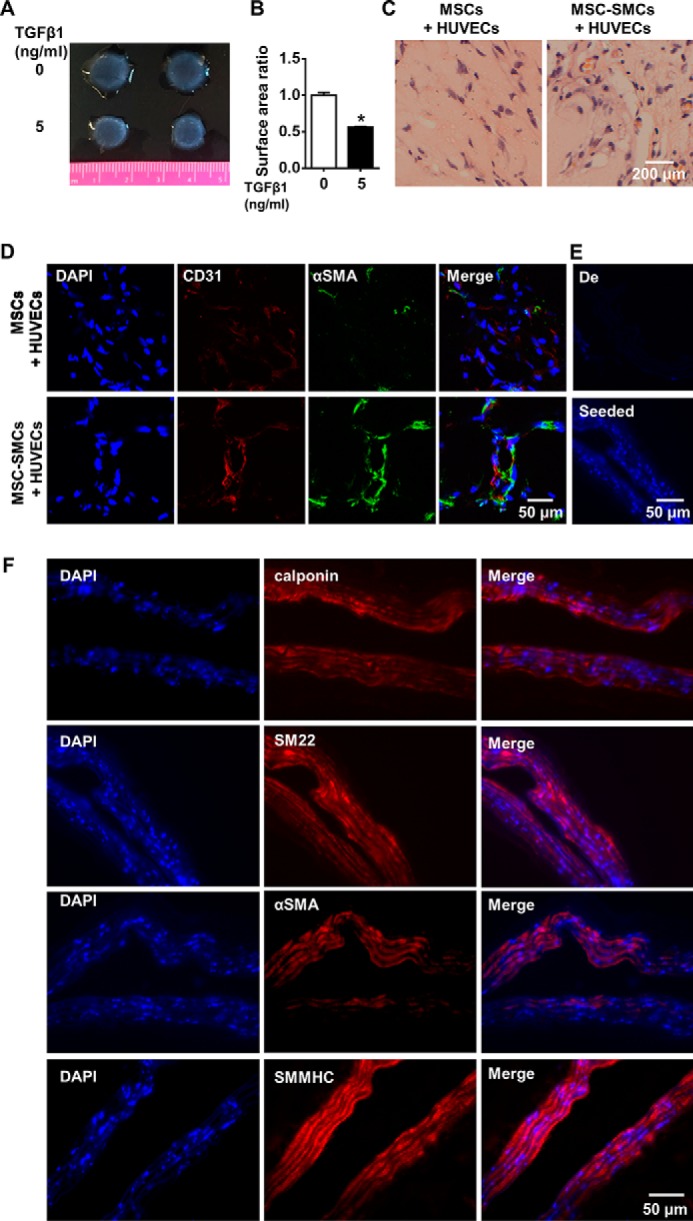
**Functional SMCs were differentiated from MSCs and gave rise to grafts with SMC layer in vascular tissue engineering.** *A* and *B,* MSCs treated with 5 ng/ml TGFβ1 displayed better contractility as shown in the representative picture from collagen I contraction assay (*A*) and correspondent statistical analysis from three independent experiments (*B*). *C,* in subcutaneous Matrigel plug assay, SMCs differentiated from mesenchymal stem cells (*MSC-SMCs*) mixed with umbilical vein endothelial cells gave rise to better vascular-like structure compared with undifferentiated mesenchymal stem cells (*MSCs*) mixed with endothelial cells as shown by H & E staining. *D,* Matrigel plugs with SMCs differentiated from mesenchymal stem cells mixed with endothelial cells showed stronger intensity of CD31 and αSMA as well as tube-like structure as demonstrated by immunofluorescent staining. Three Matrigel plugs were obtained in each group. *E,* DAPI staining of decellularized mouse aorta (*De*) and decellularized mouse aorta seeded with SMCs differentiated from MSCs (*Seeded*). DAPI staining demonstrated the colonization of seeded cells in the decellularized vascular graft, whereas the decellularized vascular graft was not stained with DAPI. *F,* SMC markers (calponin, SM22, αSMA, and SMMHC) were stained in vascular graft samples engineered by seeding SMCs differentiated from MSCs on the decellularized aorta and maintained in the *ex vivo* bioreactor system for 5 days. *A* and *C–F* are representative of three independent experiments. Data are presented as the mean ± S.D. from three independent experiments. *, *p* < 0.05. M*SC-SMCs*, smooth muscle cells differentiated from mesenchymal stem cells.

To functionally characterize the MSC-SMCs, the cells were subjected to collagen I contraction assay and subcutaneous Matrigel assay in severe combined immunodeficient (SCID) mice. SMCs differentiated from MSCs with 1% FBS and 5 ng/ml TGFβ1 displayed better contracting capacity when submitted to the collagen I contraction assay compared with cells cultured in the same medium but without TGFβ1 (Fig. 2, *A* and *B*). MSCs or MSC-SMCs were then separately mixed with human umbilical vein endothelial cells (HUVECs) in Matrigel and injected subcutaneously into SCID mice for 2 weeks to evaluate their vasculogenic properties. H&E staining showed finer tubular structures in Matrigel plug group with MSC-SMCs and endothelial cells, compared with the group containing undifferentiated MSCs and human endothelial cells (Fig. 2*C*). Immunofluorescent staining of Matrigel plug sections with SMC marker αSMA and endothelial marker CD31 demonstrated the proximity of both markers, which indicated the formation of vessel-like structures (Fig. 2*D*). Taken together, the above results demonstrated that functional SMCs could be differentiated from human umbilical cord MSCs.

### Engineering of vascular graft with MSC-derived SMCs

To set up a vascular graft model, mouse aortas were harvested and decellularized first to serve as the graft scaffold. The efficiency of the decellularization was shown by DAPI staining of the graft. Decellularized vascular graft demonstrated weak staining of destructed nuclear fractions, whereas the nucleus was intact and in an undamaged shape in the cells within the normal aorta (Fig. S1). Further inspection of the SMC marker reminiscence on the cross-sections of decellularized aorta displayed minimal SMC marker staining, and in comparison, the staining of these markers on the normal aorta demonstrated much stronger signals (Fig. S1*B*). Collectively, successful decellularization of the mouse aorta was achieved. SMCs differentiated from human MSCs were suspended in Matrigel and then wrapped outside of the decellularized scaffold, which was then connected to the bioreactor system. The graft was harvested 1 week after being kept in the bioreactor system with medium flowing inside. Staining of the graft with DAPI showed the colonization of seeded cells in the decellularized scaffold compared with the scaffold with no seeded cells (Fig. 2*E*). Further examination of the generated graft with SMC marker staining, including calponin, SM22, αSMA, and SMMHC, demonstrated that the seeded cells retained SMC marker expression after they migrated inside the decellularized scaffold (Fig. 2*F*). Moreover, the recellularized graft displayed weaker but comparable levels of calponin compared with freshly isolated mouse aorta (Fig. S1*B*), demonstrating the potency of graft engineering with differentiated SMCs from MSCs.

### Identification of miRNAs involved in the MSC to SMC differentiation process

**Figure 3.**
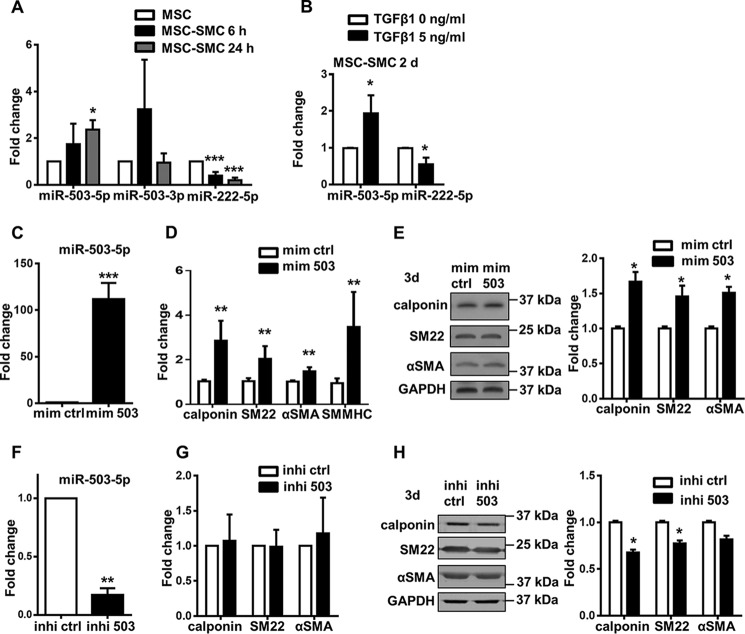
**miR-503 promotes SMC differentiation from MSCs.** *A,* level of miRNAs was detected with TaqMan microRNA assay at early time points in SMC differentiation in 1% FBS and 5 ng/ml TGFβ1. *B,* level of miRNA with or without TGFβ1 treatment after 2 days was detected with TaqMan microRNA assay. *C,* TaqMan microRNA assay showed significant up-regulation of miR-503 after mimic treatment for 1 day in αMEM with 1% FBS. *D,* Q-PCR showed the mRNA level up-regulation of SMC-specific markers after miR-503 mimic treatment for 3 days in αMEM with 1% FBS. *E,* protein expression and quantification after miR-503 mimic treatment for 3 days in αMEM with 1% FBS were analyzed. *F,* TaqMan microRNA assay showed significant down-regulation of miR-503 after inhibitor treatment for 1 day. *G,* level of SMC-specific markers was detected with Q-PCR after miR-503 inhibitor treatment for 3 days in αMEM with 1% FBS and 5 ng/ml TGFβ1. *H,* protein expression and quantification after miR-503 inhibitor treatment for 3 days in αMEM with 1% FBS and 5 ng/ml TGFβ1 were analyzed. Data are presented as the mean ± S.D. from three independent experiments. *, *p* < 0.05; **, *p* < 0.01, and ***, *p* < 0.001. *mim ctrl*, miRNA mimic negative control; *mim 503*, miR-503 mimic; *inhi ctrl*, miRNA inhibitor negative control; *inhi 503*, miR-503 inhibitor. *MSC-SMCs*, smooth muscle cells differentiated from mesenchymal stem cells.

To identify miRNAs involved in the early phase of MSC to SMC differentiation, MSCs differentiated for 6 and 24 h were harvested and subjected to miRNA array analysis with undifferentiated MSCs as control. The miRNAs up-regulated more than 1.5-fold or down-regulated more than 0.5-fold in MSC-SMCs compared with undifferentiated MSCs at either the 6-h time point or the 24-h time point were listed (Table 1). Among the up-regulated miRNAs, we identified the miR-15 family, including the stem-loop forms of miR-503 and miR-424, miR-503 mature strand miR-503-5p, and the miR-503 star strand miR-503-3p (Table 1). Changes in the expression of selected miRNAs during the differentiation process were further confirmed with TaqMan microRNA assay (Fig. 3, *A* and *B*). Consistent with the microRNA array results, the up-regulation of miR-503-5p and the down-regulation of miR-222-5p were time-dependent (Fig. 3*A*). Moreover, TGFβ1 appeared to be a major contributor to the changes in the level of those microRNAs (Fig. 3*B*), which suggests the possible involvement of these miRNAs in TGFβ1-signaling pathways. The expression level of miR-503-3p fluctuated, which compromised the stability of the expression. For this reason, miR-503-3p was not included in further experiments, and miR-503 was used to refer to miR-503-5p in the rest of the paper.

**Table 1:** **Fold change and *p* value of selected miRNAs from miRNA array** undiff, undifferentiated.

miRNAs	6 h *vs.* undiff. fold change	6 h *vs.* undiff. *p* value	24 h *vs.* undiff. fold change	24 h *vs.* undiff. *p* value
Stem loop	1.907	0.0206	2.038	0.0646
hsa-mir-424				
Stem loop	1.884	0.0041	1.263	0.1130
hsa-mir-503				
hsa-miR-503-3p	8.548	0.0063	1.422	0.3095
hsa-miR-503-5p	1.577	0.0876	2.407	0.0072
hsa-miR-145-5p	0.943	0.0920	1.522	0.0053
hsa-miR-145-3p	1.433	0.2585	1.744	0.0927
hsa-miR-143-5p	1.352	0.2608	3.668	0.0307
hsa-miR-6890-5p	1.461	0.1086	2.474	0.0420
hsa-miR-6780b-5p	0.740	0.2421	2.160	0.0147
hsa-miR-3651	1.872	0.0265	1.970	0.1528
hsa-miR-7106-5p	1.840	0.0163	1.739	0.1641
hsa-miR-6730-5p	1.334	0.1647	1.498	0.0317
hsa-miR-6813-5p	2.128	0.0072	1.215	0.4682
hsa-miR-670-5p	2.085	0.0077	1.660	0.1752
hsa-miR-222-5p	0.291	0.0018	0.093	0.0052
hsa-let-7a-2-3p	0.331	0.0049	0.365	0.0307
hsa-miR-4443	0.398	0.0204	0.348	0.0485
hsa-miR-3617-5p	0.488	0.0041	0.427	0.0029
hsa-miR-5100	0.506	0.0031	0.471	0.0179
hsa-miR-92a-1-5p	0.356	0.0537	0.228	0.0306
hsa-miR-3617-5p	0.488	0.0041	0.428	0.0029
hsa-miR-378f	0.864	0.2043	0.438	0.0494
hsa-miR-758-5p	0.424	0.0609	0.451	0.0046
hsa-miR-1303	0.702	0.2350	0.468	0.0438
hsa-miR-1260b	0.716	0.0901	0.484	0.0004
hsa-miR-4286	0.478	0.0120	0.485	0.0272
hsa-miR-548ae	0.635	0.0974	0.484	0.0454

### miR-503 promotes MSC to SMC differentiation

To explore the role of miR-503 in MSC differentiation toward SMCs, miRNA mimics and inhibitors were used to perform the gain-of-function and loss-of-function analysis of miR-503. The transfection efficiency was confirmed 24 h after transfection as shown by a more than 100-fold increase of miR-503 expression in MSCs transfected with the miR-503 mimics (Fig. 3*C*). Transfection of miR-503 mimics in MSCs in medium with 1% FBS promoted SMC differentiation with increased expression of SMC markers, including calponin, SM22, αSMA, and SMMHC at the mRNA level after 3 days as shown by Q-PCR (Fig. 3*D*). Up-regulation of calponin, SM22, and αSMA was further confirmed by their increased protein expression with Western blot analysis (Fig. 3*E*). In addition, promotion of SMC differentiation was also observed in human adipose tissue-derived MSCs (Fig. S2, *A* and *B*) and mouse adipose tissue-derived MSCs (Fig. S2, *C–E*), which is demonstrated by the induction of SMC markers with miR-503 mimic treatment at the mRNA and protein level. Loss-of-function effects of miR-503 were demonstrated by the transfection of miR-503 inhibitors in the cells. One day after transfection, the level of miR-503 was significantly decreased (Fig. 3*F*). SMC markers, including calponin, SM22, and αSMA, were not altered in mRNA expression as shown by Q-PCR (Fig. 3*G*). However, a moderate reduction of the expression of these SMC markers could be observed at the protein level (Fig. 3*H*). This suggested that some other post-transcriptional regulation processes influencing mRNA translation and protein stability might exist (19).

### SMAD7 is a direct target of miR-503 and miR-503 is transcriptionally up-regulated through SMAD4-dependent pathway

**Figure 4.**
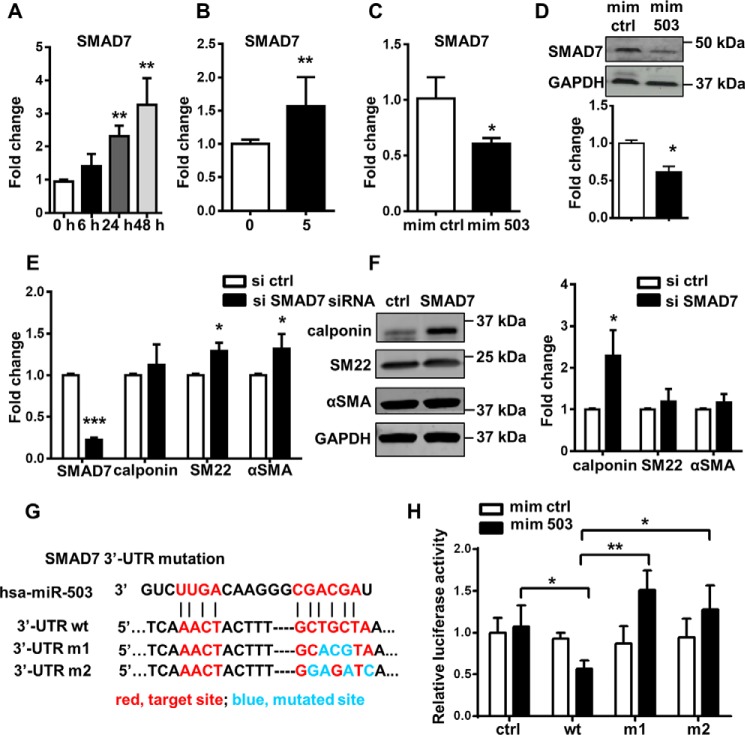
**miR-503 directly targets SMAD7 in regulating SMC differentiation.** *A–C,* SMAD7 mRNA was detected with Q-PCR at different time points during SMC differentiation in 1% FBS and 5 ng/ml TGFβ1 (*A*), after treatment with TGFβ1 for 2 days compared with cells cultured only in 1% FBS (*B*), and after treatment with miR-503 mimics in αMEM with 1% FBS for 3 days (*C*). *D,* representative picture of Western blotting and analysis from three independent experiments showed the down-regulation of SMAD7 at the protein level after treatment with miR-503 mimics in αMEM with 1% FBS for 3 days. *E,* Q-PCR of SMAD7 and SMC markers after siRNA transfection for 2 days in αMEM with 1% FBS. *F,* Western blotting image and analysis after siRNA transfection for 2 days in αMEM with 1% FBS. Representative image was shown from three independent experiments. *G,* alignment of miR-503 and the 3′-UTR of *SMAD7* gene showed the postulated target-binding sites (*red*) and induced mutations (*blue*). Two different kinds of mutations were induced (m1 and m2) of the same site. *H,* co-transfection of miR-503 mimics and reporter with WT SMAD7 3′-UTR segment showed reduced relative luciferase activity as compared with vector with empty plasmid, whereas mutation of target-binding sites recovered the reduction. Relative luciferase activity was calculated with firefly luciferase activity/*Renilla* luciferase activity. Data are presented as the mean ± S.D. from three independent experiments. Statistics were obtained from two-way ANOVA test followed by Bonferroni post hoc analysis. *, *p* < 0.05; **, *p* < 0.01; and ***, *p* < 0.001. *mim ctrl,* miRNA mimic negative control; *mim 503*, miR-503 mimic; *si ctrl,* siRNA negative control; *si SMAD7,* siRNA SMAD7; *ctrl,* plasmid negative control; *wt,* plasmid bearing WT SMAD7 3′-UTR.

To explore the potential targets of miR-503, algorithm-based bioinformatic prediction, literature review, and *in vitro* examination of gene expression were conducted. TargetScan was used for web-based target prediction (20). After obtaining the list of predicted targets, further screening was performed to select targets related to TGFβ1-signaling pathway, given the importance of TGFβ1 in SMC differentiation and its role in regulating miR-503 expression. Genome browsing in the UCSC genome sequence database (21) implied that the 3′-UTR of SMAD7 contain a “GCTGCTA” sequence that may be a target site for miR-503. SMAD7 exhibits an inhibitory effect on SMAD-dependent signaling pathway by binding to the TGFβ type I receptor on the cell membrane, thus blocking the binding of the R-SMAD–co-SMAD complex to the receptor or the phosphorylation of the complex (22). SMAD7 mRNA expression was up-regulated in a time-dependent manner during the MSC to SMC differentiation process (Fig. 4*A*) When comparing MSC-SMCs differentiated in medium with or without TGFβ1, it was observed that the SMAD7 up-regulation could be attributed to TGFβ1 treatment (Fig. 4*B*).

More importantly, the level of SMAD7 upon miR-503 mimic treatment showed significant down-regulation by Q-PCR (Fig. 4*C*). Western blot analysis confirmed the result obtained from Q-PCR at the protein level (Fig. 4*D*). Loss-of-function study by siRNA knockdown experiments showed that loss of SMAD7 (Fig. 4*E*) resulted in the up-regulation of SMC markers SM22 and αSMA at the mRNA level (Fig. 4*E*). At the protein level, only calponin was significantly up-regulated (Fig. 4*F*), which implied the existence of other post-translational or compensating mechanisms. Ultimately, to explore whether there is direct inhibition of miR-503 on SMAD7, the SMAD7 3′-UTR segment was cloned into an miRNA target reporter clone with firefly luciferase gene located upstream of the SMAD7 3′-UTR, and the target sites at SMAD7 3′-UTR predicted with TargetScan were then mutated with the QuickChange Lightning site-directed mutagenesis kit (Agilent Technologies) (Fig. 4*G*). It was revealed that miR-503 co-transfection in HEK293 cells could inhibit the relative luciferase activity in plasmid reporter with SMAD7 3′-UTR compared with miRNA control in plasmid reporter with SMAD7 3′-UTR, and the inhibition was abolished if the miR-503 target site on the 3′-UTR segment was mutated (Fig. 4*H*). *Renilla* luciferase served as an internal control of the plasmid transfection efficiency.

**Figure 5.**
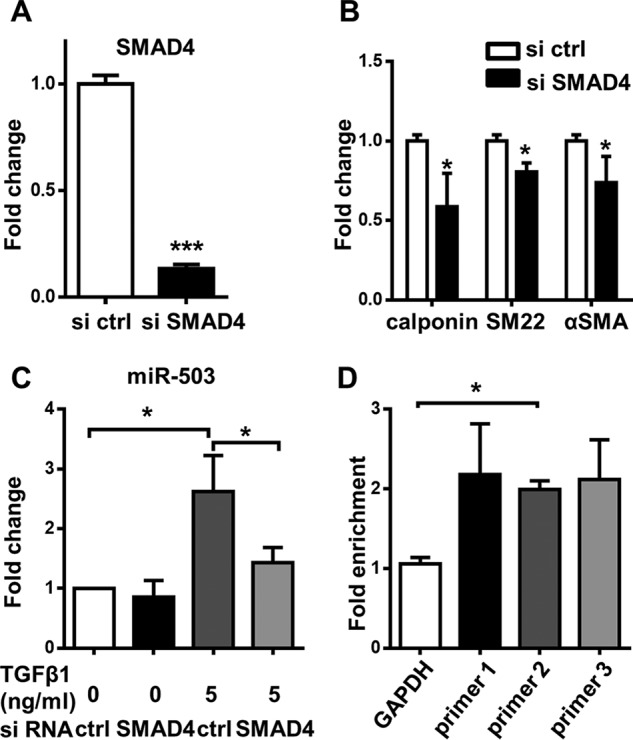
**miR-503 is transcriptionally up-regulated through SMAD4-dependent pathway.** *A,* SMAD4 mRNA was detected with Q-PCR after treatment with siRNA for 1 day in αMEM with 1% FBS and 5 ng/ml TGFβ1. *B,* Q-PCR of SMC markers after siRNA transfection for 2 days in αMEM with 1% FBS and 5 ng/ml TGFβ1. *C,* level of miR-503 detected with TaqMan microRNA assay after cells were treated with siRNA with or without TGFβ1 for 2 days. *D,* MSCs were starved and then treated with or without TGFβ1 for 4 h and then harvested for ChIP experiments. Cells cultured without TGFβ1 were used as a control. Three primers (primer 1, primer 2, and primer 3) specific to the miR-503 promoter region were used to detect the enrichment of SMAD4. A primer specific to the GAPDH promoter region was used as a negative control. Fold enrichment was calculated against input and the control without TGFβ1 treatment. Data were obtained from at least three independent experiments and shown as mean ± S.D. Statistics were obtained with *t* test (*A* and *B*) or one-way ANOVA (*C* and *D*), followed by Bonferroni post hoc analysis. *, *p* < 0.05, and ***, *p* < 0.001.

By establishing SMAD7 as a direct target of miR-503, we present miR-503 as a new component of the TGFβ1-signaling pathway. As demonstrated earlier, SMAD7 and miR-503 are both transcriptionally up-regulated by TGFβ1. A possible explanation is that miR-503 could be up-regulated by the same machinery that up-regulates SMAD7 and could serve as a limiting factor to fine-tune its up-regulation. To test this hypothesis, knockdown experiments of SMAD4 were performed because SMAD4 is a co-SMAD factor necessary for all SMADs to function and participates in SMAD7 up-regulation. SMAD4 knockdown was confirmed by the significant decrease of SMAD4 expression and downstream SMC markers, including calponin, SM22, and αSMA (Fig. 5, *A* and *B*). Next, the level of miR-503 was examined after SMAD4 knockdown. Interestingly, miR-503 was significantly down-regulated when SMAD4 was depleted in cells with TGFβ1, although its level was not affected if the medium did not contain TGFβ1 (Fig. 5*C*). Interestingly, SMAD7 is also up-regulated in a SMAD4-dependent pathway suggesting that miR-503 might serve as a self-limiting factor for the SMAD4-dependent induction of SMAD7 (Fig. S3). These results implied that SMAD4 plays a key role in TGFβ1-mediated up-regulation of miR-503. We then performed chromatin immunoprecipitation (ChIP) experiments to detect SMAD4 binding, and three sets of primers were used to target the promoter region of miR-503. Enrichment of SMAD4 at the promoter region of miR-503 was confirmed, and at the same time SMAD4 was not enriched at the promoter region of GAPDH, which served as a negative control (Fig. 5*D*). Taken together, we showed that miR-503 is transcriptionally up-regulated upon TGFβ1 treatment through a SMAD4-dependent pathway and subsequently targets SMAD7, which is a negative regulator of SMAD-dependent signaling.

### miR-222-5p inhibits SMC differentiation

miR-222-5p showed the greatest decrease in the MSC to SMC differentiation process as revealed by miRNA array and subsequent confirmation with TaqMan microRNA assay (Table 1, Fig. 3, *A* and *B*). miR-222, which belongs to the same miRNA family as miR-221, was identified as an important modulator in platelet-derived growth factor–induced SMC phenotypic change (23). However, only the mature strand was examined in the study, whereas the down-regulated miR-222-5p during SMC differentiation in our experimental system is the passenger strand, in which role has not yet been established. Although the passenger strand of miR-222 (*i.e.* miR-222-5p) was widely accepted to be destined for degradation without any function after maturation, recent studies have begun to depict the pathological importance of passenger strands of miRNAs (24, 25).

**Figure 6.**
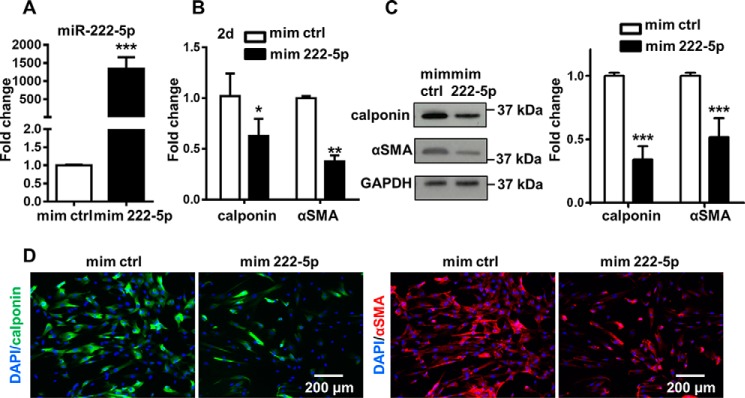
**miR-222-5p inhibits SMC differentiation from MSCs.** *A,* level of miR-222-5p was detected with TaqMan microRNA assay. *B,* Q-PCR showed the gene expression of SMC markers (calponin and αSMA) after cells were treated with miRNA mimics in αMEM with 1% FBS and 5 ng/ml TGFβ1 for 2 days. *C,* representative Western blotting image and analysis after cells were treated with miRNA mimics in αMEM with 1% FBS and 5 ng/ml TGFβ1 for 2 days were obtained from three independent experiments. *D,* immunofluorescent staining showed the intensity of SMC markers (calponin and αSMA) after miR-222-5p treatment for 2 days. Cell nucleus was stained with DAPI (*blue*). Representative images were obtained from three independent experiments. Data were obtained from at least three independent experiments and shown as mean ± S.D. *, *p* < 0.05; **, *p* < 0.01; and ***, *p* < 0.001. *mim ctrl*, miRNA mimic negative control; *mim 222-5p*, miR-222-5p mimic.

The influence of miR-222-5p on the differentiation process was first investigated by transfecting miR-222-5p mimics into MSCs. The success of miRNA mimic transfection was confirmed by the significant increase of miR-222-5p levels (Fig. 6*A*). Increased level of miR-222-5p prompted the down-regulation of SMC markers, including calponin and αSMA both in the mRNA expression by Q-PCR (Fig. 6*B*) and at the protein level by Western blotting and immunofluorescent staining (Fig. 6, *C* and *D*). Moreover, similar results have been observed in human adipose tissue-derived MSCs (Fig. S4, *A* and *B*). The results above demonstrated the capacity of miR-222-5p mimics in inhibiting SMC differentiation. Induction of SMC markers was also shown with miR-222-5p inhibitor treatment; however, the effect is moderate (Fig. S5), possibly due to the already significantly reduced level of miR-222-5p in the differentiation process.

### 3′-UTR segments of ROCK2 and αSMA are direct targets of miR-222-5p

In addition to SMAD-dependent TGFβ1-mediated pathways, SMC differentiation is also promoted through the RhoA/ROCK pathway (RhoA is ras homolog gene family, member A) (26, 27). Thus, components of both pathways were examined for potential miR-222-5p targets. 3′-UTR sequences of selected genes were subjected to screening for the putative seed site of miR-222-5p, “CTACTGA.” It was revealed that ROCK2 contains two putative seed sites that strongly implied that it might be directly targeted by miR-222-5p.

**Figure 7.**
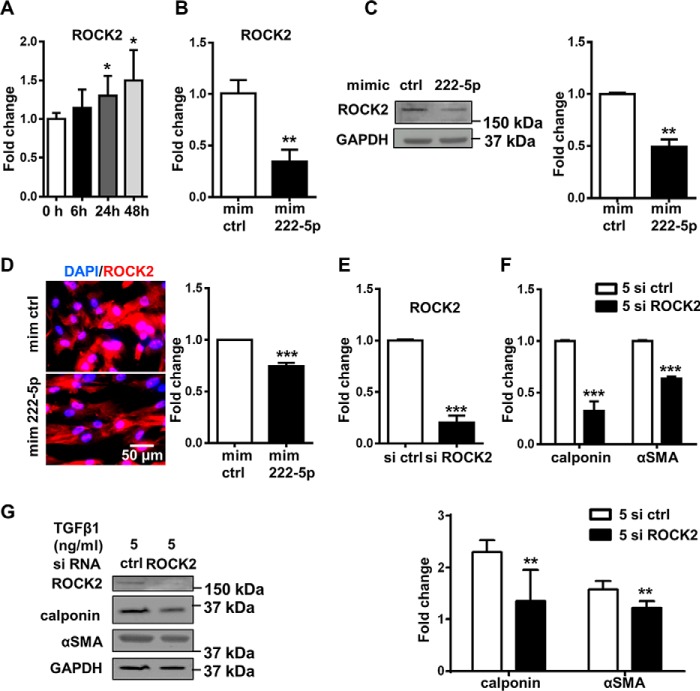
**ROCK2 3′-UTR is a potential target of miR-222-5p.** *A,* Q-PCR showed the up-regulation of ROCK2 in a time-dependent manner during SMC differentiation in 1% FBS and 5 ng/ml TGFβ1. *B,* miR-222-5p mimic treatment in αMEM with 1% FBS and 5 ng/ml TGFβ1 for 2 days inhibited the level of ROCK2 mRNA as shown by Q-PCR. *C,* Western blotting and analysis of ROCK2 after miR-222-5p mimic treatment in αMEM with 1% FBS and 5 ng/ml TGFβ1 for 2 days. *D,* protein level of ROCK2 (*red*) was detected with immunofluorescent staining and correspondent analysis. DAPI (*blue*) was used to stain the nucleus. *E,* efficiency of siRNA ROCK2 was confirmed with significant down-regulation of ROCK2 detected with Q-PCR. *F,* Q-PCR showed the down-regulation of SMC markers after treatment of siRNA ROCK2 in αMEM with 1% FBS and 5 ng/ml TGFβ1 for 2 days. *G,* Western blotting and analysis of cells treated with siRNA in medium with TGFβ1. Images shown (*C, D,* and *G, left panels*) were representative of three independent experiments. Data were obtained from at least three independent experiments and shown as mean ± S.D. *, *p* < 0.05; **, *p* < 0.01, and ***, *p* < 0.001. *mim ctrl,* miRNA mimic negative control; *mim 222-5p*, miR-222-5p mimic, *si ctrl*, siRNA negative control; *si ROCK2*, siRNA ROCK2.

The expression of ROCK2 during MSC-SMC differentiation was analyzed, and Q-PCR demonstrated an up-regulation of ROCK2 mRNA expression in a time-dependent manner (Fig. 7*A*). Next, the ROCK2 level was investigated after miR-222-5p mimic treatment. Q-PCR analysis showed the down-regulation of ROCK2 mRNA expression 1 day after miR-222-5p mimic treatment (Fig. 7*B*). Inhibition of ROCK2 at the protein level was demonstrated by Western blot analysis and immunofluorescent staining (Fig. 7, *C* and *D*). ROCK2 was also inhibited at the mRNA level with miR-222-5p mimic treatment in human adipose tissue-derived MSCs (Fig. S4*C*). Taken together, ROCK2 expression increased in a time-dependent manner during differentiation and could be inhibited by miR-222-5p mimic treatment. Knockdown of ROCK2 with siRNA was then performed to confirm the importance of ROCK2 in MSC to SMC differentiation. ROCK2 knockdown was confirmed at both gene expression and protein levels (Fig. 7, *E* and *G*). Subsequent examination of SMC markers revealed significant inhibition of SMC markers, including calponin and αSMA, both in mRNA expression and at the protein level (Fig. 7, *F* and *G*).

**Figure 8.**
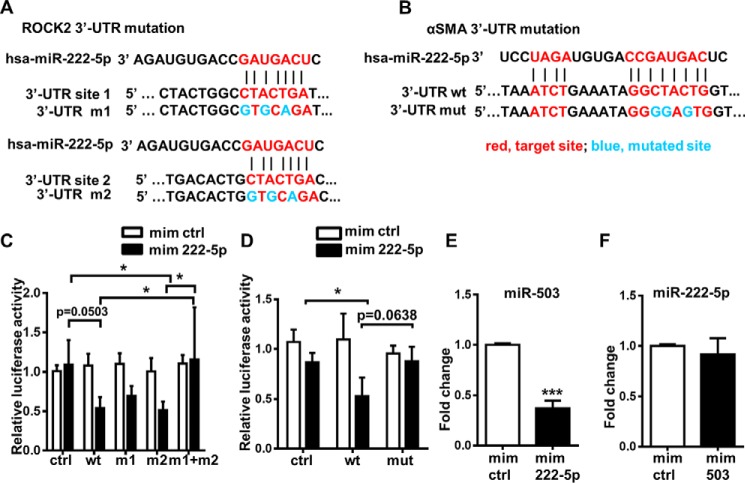
**3′-UTRs of ROCK2 and αSMA were direct targets of miR-222-5p.** *A,* alignment of miR-222-5p and ROCK2 3′-UTR showed the postulated target-binding sites (*red*) and induced mutations (*blue*). ROCK2 3′-UTR contains two target-binding sites (*site 1* and *site 2*) of miR-222-5p, which were mutated alone (*m1, m2*) or together (*m1*+*m2*). *B,* alignment of miR-222-5p and the 3′-UTR of αSMA showed the postulated target-binding sites (*red*) and induced mutations (*blue*). *C,* co-transfection of miR-222-5p mimics and reporter plasmid with WT ROCK2 3′-UTR showed reduced relative luciferase activity as compared with vector with empty plasmid, whereas mutation of both target-binding sites (*m1*+*m2*) recovered the reduction. *D,* dual transfection of plasmids and miR-222-5p into HEK293 cells demonstrated the inhibition of miR-222-5p on the 3′-UTR of αSMA, and mutation of the predicted target site recovered the inhibition. Relative luciferase activity was calculated with firefly luciferase activity/*Renilla* luciferase activity. Data are presented as the mean ± S.D. from three independent experiments. Statistics (*C* and *D*) were obtained from two-way ANOVA test followed by Bonferroni post hoc analysis. *E,* level of miR-503 was inhibited by miR-222-5p mimic treatment after 1 day as shown with TaqMan microRNA assay. *F,* TaqMan microRNA assay of miR-222-5p did not reveal any change after miR-503 mimic treatment. Data are presented as the mean ± S.D. from three independent experiments. *, *p* < 0.05, and ***, *p* < 0.001. *mim ctrl,* miRNA mimic negative control; *mim 503,* miR-503 mimic; *ctrl,* plasmid without SMAD7 3′-UTR; *wt,* plasmid bearing WT SMAD7 3′-UTR.

After establishing that miR-222-5p mimics decreased ROCK2 expression and ROCK2 played a functional role in promoting SMC differentiation, we explored whether miR-222-5p could inhibit ROCK2 through direct targeting of ROCK2 3′-UTR. The 3′-UTR segment of ROCK2 was therefore cloned into miRNA target reporter plasmids. The two putative target sites within the 3′-UTR segment were mutated separately or simultaneously to understand whether the inhibition occurred through the complementary binding to single or both putative target sites (Fig. 8*A*). WT and mutated plasmids were transfected into HEK293 cells. Results showed that, compared with the empty plasmid, the plasmid containing the WT ROCK 3′-UTR demonstrated lower relative luciferase activity when co-transfected with miR-222-5p mimics, and this effect was not observed when the plasmids were co-transfected with miRNA mimic negative control (Fig. 8*C*). Mutation of both target sites, but not a single target site, rescued the inhibited relative luciferase activity (Fig. 8*C*). Therefore, we concluded that ROCK2 3′-UTR is a direct target of miR-222-5p.

Furthermore, given the prominent inhibitory effect of miR-222-5p mimics on αSMA expression both at the gene expression and the protein levels, it was postulated that αSMA 3′-UTR might be directly targeted by miR-222-5p. To test this hypothesis, the sequence of miR-222-5p was aligned with αSMA mRNA, and complementarity was found at both the 5′- and 3′-end of miR-222-5p with the 3′-UTR of αSMA mRNA (Fig. 8*B*). Luciferase reporter assays with insertions of either αSMA 3′-UTR sequence or mutated sequence at predicted complementary sites were then utilized to validate whether 3′-UTR of αSMA mRNA could be directly targeted by miR-222-5p. Dual transfection of the plasmid encoding αSMA 3′-UTR sequence and miR-222-5p mimics into HEK293 cells revealed that miR-222-5p mimics inhibited αSMA 3′-UTR, and the mutation of the target site within the 3′-UTR rescued the inhibition to a certain degree (Fig. 8*D*). Collectively, the evidence implies that miR-222-5p could directly target αSMA 3′-UTR and lead to down-regulation of αSMA both at the gene expression and the protein level.

### Interaction of miR-503 and miR-222-5p

Thus far, we established that the up-regulation of miR-503 and the down-regulation of miR-222-5p both regulate MSC to SMC differentiation. Although they target different pathways regulating SMC differentiation, whether miR-503 and miR-222-5p directly interact with each other merited further examination. The lack of complementary sequence between miR-503 and miR-222-5p suggests that they are unlikely to directly bind to each other (data not shown). However, 24 h after transfection of the miR-222-5p mimic in MSCs, the level of miR-503 was significantly down-regulated as shown by TaqMan microRNA assay (Fig. 8*E*). On the contrary, 24 h after transfection of miR-503 mimic in MSCs, the level of miR-222-5p was not affected (Fig. 8*F*). This suggested that miR-222-5p could affect the expression of miR-503, but miR-503 does not interfere with the expression of miR-222-5p.

## Discussion

Recently, significant progress in vascular tissue engineering has been made to produce small-diameter vessel grafts for clinical applications (28). Several sources of stem cells that can differentiate into SMCs have been employed (29). However, there are limitations in terms of obtaining a sufficient number of cells or the risk of tumor formation. MSCs from human umbilical cord could be a novel source of SMCs because they display very low alloimmune response with a low risk of tumorigenicity (30–32). In this study, we have successfully differentiated these stem cells into the vascular smooth muscle lineage. We also described the differentiation mechanism that involves miRNAs. Numerous miRNAs are involved in SMC differentiation (15), some of which are induced by TGFβ1 such as miR-143/145 (16) and miR-21 (33). In our system, miR-143/145 were up-regulated (Table 1); however, miR-21 was not altered (data not shown). Explanation might lie in the highly context-dependent effect of miR-21 (34).

It was established in our study that miR-503 targets SMAD7 to promote MSC to SMC differentiation, and miR-222-5p targets ROCK2 to inhibit the differentiation process. Moreover, we showed that SMAD4 is enriched at the promoter region of miR-503 upon TGFβ1 treatment. We also showed that miR-503 was regulated by miR-222-5p. Thus, we provided the first evidence that MSCs from human umbilical cord can generate functional SMCs through miRNA regulation.

The miR-503 family participates in a number of pathophysiological pathways (35). It was shown that miR-424, which belongs to this family, can target cyclin D1 to decrease SMC proliferation and ultimately attenuate neointimal formation after vascular injury (35). In our study, miR-503 directly targets SMAD7 by binding to its 3′-UTR segment. This is the first time that miR-503 has been shown to target SMAD7, thereby influencing the SMC differentiation process. Importantly, miR-503 could also promote SMC differentiation in other types of MSCs, suggesting its universal effect among MSCs. In the SMAD-dependent signaling pathway, TGFβ1 binds to and activates the TGFβ receptors on the cell membrane, and it subsequently mediates the phosphorylation of the R-SMAD–co-SMAD complex. The phosphorylated complex is then translocated to the nucleus and binds to the SMAD-binding element at the promoter region of target genes, thereby up-regulating the gene transcription (36). Interestingly, although SMAD7 is a well-known negative regulator of the TGFβ1 SMAD-dependent signaling pathway, we showed that it could be transcriptionally up-regulated by TGFβ1, suggesting a negative feedback loop control for TGFβ1 signaling (22, 37). The inhibition of SMAD7 by miR-503 provides a further level of complexity in the already complex TGFβ1-related signaling pathway. However, the augmented SMC differentiation after siRNA-mediated SMAD7 knockdown was mainly exhibited on calponin at the protein level, suggesting that other mechanisms mediating SMC differentiation might also be at play. Previous studies related to the upstream regulation of miR-503 transcription have described peroxisome proliferator-activated receptor γ and nuclear factor κ–light-chain enhancer of activated B cells (NF-κB) as direct regulators (38–40). In this study, SMAD4 enriches at the promoter region of miR-503 after TGFβ1 treatment.

miR-222 participates in numerous physiological and pathophysiological conditions, including cancer progression (41), skeletal muscle regeneration (42), and vascular remodeling (43). However, to date, there is no report available on the direct relation between miR-222 and the TGFβ1-signaling pathway. In addition, the mature strand miR-222-3p was examined in most studies. In our stem cell to SMC differentiation system, miR-222-5p, which is the passenger strand of miR-222, was examined. Accumulating reports have emerged in recent years to depict the fate of miRNA passenger strand as well as their regulatory role in human diseases such as cancers, inflammatory diseases, and cardiovascular diseases (44–47). The gain-of-function assessment with the miRNA mimic demonstrated their prominent role in inhibiting SMC differentiation. The down-regulation of this miRNA might serve as a prerequisite for the up-regulation of contractile SMC markers in the differentiation process. During SMC differentiation, miR-222-5p down-regulation might work together with miR-503 up-regulation.

RhoA/ROCK pathway plays a crucial role in various biological processes, including cellular migration and differentiation. In addition to the SMAD-dependent signaling pathway, RhoA/ROCK pathway could also be activated upon TGFβ1 stimulation and participate in SMC differentiation (48). There are two isoforms of ROCK, including ROCK2, that are mainly expressed in SMCs. ROCK2 was shown to be a direct target of miR-222-5p in our study. Furthermore, target site mutation experiments confirmed that αSMA 3′-UTR might be a direct target of miR-222-5p. However, the mutation of the target site complementary to the 5′-end of the miRNA did not consistently recover the inhibition of the miR-222-5p on plasmids containing αSMA 3′-UTR, implying that the site complementary to the 3′-end might also have some effect in inducing the complementary binding of miR-222-5p to αSMA 3′-UTR and promoting the inhibitory effect.

Multiple TGFβ1-related signaling pathways interact with each other and work synergistically. It was also found that the RhoA/ROCK-signaling pathway and SMAD-dependent–signaling pathway could regulate each other (49, 50). Some miRNAs share similarity or display complementarity in sequence with other miRNAs and thus demonstrate interactions with each other (51–53). Furthermore, some miRNAs were shown to indirectly control the transcription of other miRNAs (54, 55). Although the miRNA interaction network has been gradually recognized, large scale data pool and subsequent bioinformatic analyses would be required to understand the complexity of the regulation network among multiple miRNAs, which could not be realistically achieved in the experimental system of this study. Nonetheless, we found that one miRNA might exert an effect on another. miR-222-5p mimic transfection resulted in miR-503 down-regulation. The postulation that downstream targets of miR-222-5p may also regulate miR-503 expression would merit further examination.

In this study, SMCs were differentiated from MSCs of human umbilical cord and exhibited the potential for engineering vascular grafts. The mechanistic study involving miRNAs provides novel opportunities to improve graft performance. SMCs pretreated with miR-29a inhibitors for vascular tissue engineering performed better than cells treated with TGFβ1 in producing elastin, which is a prominent component of extracellular matrix and contributes significantly to the mechanical strength of the engineered vessel (56). The implication from our *in vitro* study is that miR-503 mimics and miR-222-5p inhibitors may have the potential to augment the performance of vascular grafts by promoting the differentiation of stem cells toward SMCs.

**Figure 9.**
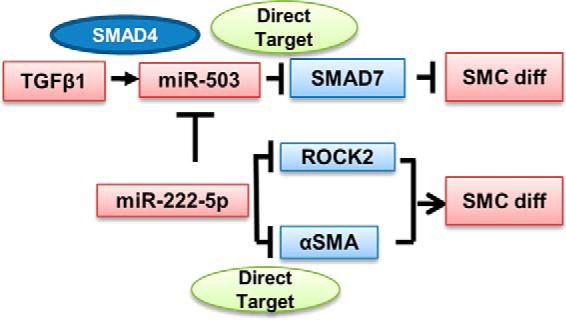
**Schematic graph of miRNA-involved pathways in the SMC differentiation process from MSCs.** In MSC to SMC differentiation, upon stimulation of TGFβ1, miR-503 is up-regulated in a SMAD4-dependent pathway and directly targets SMAD7, which is a negative regulator of the TGFβ1 SMAD-dependent signaling pathway, to promote SMC differentiation. The level of miR-222-5p was down-regulated in the differentiation process. This results in de-repression of its direct targets ROCK2 and αSMA and subsequent promotion of SMC differentiation. Furthermore, the expression of miR-503 could be inhibited by miR-222-5p.

In summary, this study has achieved the initial aims of inducing the differentiation of SMCs from human umbilical cord MSCs and of elucidating the underlying mechanisms (schematic graph of the miRNA-involved SMC differentiation mechanism is illustrated in Fig. 9). Briefly, umbilical cord MSCs were demonstrated to have the potential to differentiate toward SMCs, and MSC-derived SMCs had the ability to generate the smooth muscle layer of vascular grafts. Furthermore, the mechanistic study implied that miR-503 mimics or miR-222-5p inhibitors carry the potential to improve the performance of these vascular grafts through enhancing SMC differentiation from MSCs while avoiding possible off-target effects from the use of TGFβ1. Future studies are needed to elucidate the therapeutic potential of miRNAs and to explore the interaction of multiple signaling pathways, which could lead to refined approaches for the generation of tissue-engineered vascular grafts for clinic applications.

## Experimental procedures

### Cell culture of umbilical cord-derived MSCs

MSCs of human umbilical cord were purchased from the ATCC (PCS-500-010) and were cultured on flasks coated with 0.04% gelatin (diluted from 2% gelatin, Sigma G1393) in ATCC mesenchymal stem cell basal medium (PCS-500-030) supplemented with one kit of mesenchymal stem cell growth kit-low serum (PCS-500-040) and 100 units/ml penicillin and streptomycin in a humidified incubator supplemented with 5% CO_2_. Complete MSC culture medium contained 2% FBS, 5 ng/ml rh FGF basic, 5 ng/ml rh FGF acidic, 5 ng/ml rh EGF, and 2.4 mm l-alanyl–l-glutamine. Cells were passaged every other day at the ratio of 1:2. Cells below passage 15 were used for all experiments.

### Smooth muscle differentiation

For SMC differentiation, the differentiation medium was αMEM with 1 or 10% FBS and 0 or 5 ng/ml TGFβ1 as specified in each experiment. The culture time and seeding density varied as mentioned in the text. To prevent the cells from being too dense, the seeding density is carefully controlled to ensure that the cell density is around 80% upon harvesting.

### RNA extraction, RT-PCR, and Q-PCR

Total RNA was extracted using the RNeasy mini kit (Qiagen, 74106) following the kit instructions. Reverse transcription of RNA was performed with the QuantiTect reverse transcription kit (Qiagen, 205314). 1000 ng of total RNA was used for each reaction of reverse transcription. Expression of mRNA in cells was detected by Q-PCR using Eppendorf Mastercycler ep realplex in duplicates. The primers used are listed in Table S1. Fold change of gene of interest was calculated by the threshold cycle (*C_y_*) value difference against internal control GAPDH.

Extraction of microRNA was performed with miRNeasy mini kit (Qiagen, 217004) following the kit instructions. Reverse transcription of miRNA was performed with microRNA reverse transcription kit (Life Technologies, Inc., 4366597) according to the manual. Specific TaqMan microRNA assay and TaqMan Master Mix (Life Technologies, Inc., 4440040) were used to assess the expression of microRNAs. All samples were run in duplicates and standardized to U6.

### Western blotting

RIPA buffer (Life Technologies, Inc., 89901) with phosphatase inhibitor and protease inhibitor (Roche Applied Science) was used to lyse cells. Cell lysates were sonicated with a Branson sonifier. Protein concentration was measured with protein assay (Bio-Rad) with Spectrophotometer 3000 (Bio-Rad) in duplicates. 10–50 μg of protein was loaded in NuPAGE 4–12% BisTris gel for protein fractionation, and protein was then transferred to nitrocellulose membrane (VWR, 732-3031). After blocking, protein was probed with primary antibody followed by detection with horseradish peroxidase-conjugated secondary antibody (Dako) after washing. The following primary antibodies were used: calponin (ab46794, Abcam); SM22 (ab14106, Abcam); αSMA (A2547-100, Sigma); SMMHC (ab53219, Abcam); SMAD7 (MAB2029, R&D Systems); and ROCK2 (ab71598, Abcam).

### Immunofluorescent staining

After the cells were cultured on the slides (Merck Millipore) for the intended time with the intended treatment with medium change every 2nd day, the cells were fixed with 4% paraformaldehyde after reaching 80% confluency. Permeabilization was then performed with 0.1% Triton X-100. For the frozen sections of vessels harvested from *ex vivo* experiments, the samples were fixed with 100% cold acetone. After fixation and permeabilization, all slides were blocked with 5% swine serum. The primary antibodies for incubation include the following: calponin (ab46794, Abcam); SM22 (ab14106, Abcam); αSMA (C6198–2ML, Sigma); SMMHC (ab53219, Abcam); and ROCK2 (ab71598, Abcam). After washing, the slides were incubated with Alexa Fluor-conjugated secondary antibodies (Life Technologies, Inc.) at 37 °C for 45 min. Slides were washed before staining with DAPI (Sigma) followed by mounting with mounting media (Dako, S3023). Images were taken with Axio Imager M2 microscope and Volocity software.

### Array of miRNA expression profile

GeneChip® miRNA 2.0 Array (Affymetrix) was used to determine the microRNA expression profile during differentiation. RNA (including microRNA) was extracted from undifferentiated MSCs, and cells were cultured in differentiation medium for 6 and 24 h. Samples were then subjected to miRNA array analysis. U6 was used as control. Fold change of miRNAs was calculated against undifferentiated cells.

### Transient transfection of miRNA mimics, miRNA inhibitors, and siRNAs

MSCs were transfected with miRNA mimics, miRNA inhibitors, or siRNAs upon confluence of 60–70% after being seeded in αMEM with 1% FBS for 24 h. Lipofectamine RNAiMAX (Invitrogen) was used for transfection. Reagents used were as follows: miRNA mimic negative control (4464058, Life Technologies, Inc.); miR-503 mimic (4464066 MC10378, Life Technologies, Inc.); miR-222-5p mimic (4464066 MC12656, Life Technologies, Inc.); miRNA inhibitor negative control (199006-001, Exiqon); miR-503 inhibitor (4100899-001, Exiqon); siRNA negative control (AM4611, Life Technologies, Inc.); siRNA SMAD7 (AM16708 155241, Life Technologies, Inc.); siRNA ROCK2 (AM51331 110867, Life Technologies, Inc.); RNAiMax (13778-075, Life Technologies, Inc.); (31985062, Opti-MEM, Life Technologies, Inc.). After optimization of concentration, the final concentration of siRNA and miRNA mimic was 12.5 nm and for miRNA inhibitor the final concentration was 60 nm.

### Luciferase reporter assays

miRNA target specificity was examined by luciferase reporter assays. A plasmid containing the 3′-UTR of target genes was cloned downstream of humanized firefly luciferase, and synthetic *Renilla* luciferase gene was included in the plasmid for indication of plasmid transfection efficiency. Plasmids are from Genecopoeia. 1.5 × 10^4^ HEK293 cells were seeded in each well of 24-well plates and transfected the next day upon the density of 60–70%. Lipofectamine 3000 (L3000015, Life Technologies, Inc.) was used for transfection. Firefly and *Renilla* luciferase activity was detected with luciferase assay kit (Promega) 24 h after transfection.

### Site mutation of plasmids

Mutation of the target site in plasmids was performed with QuickChange Lightning site-directed mutagenesis kit (210518, Agilent Technologies) following the manual. Primers for mutation of the target site was designed with the website https://www.genomics.agilent.com/primerDesignProgram.jsp^5^ based on the intended mutation. Primers used for each 3′-UTR mutation are listed in Table S2. Amplified plasmids were extracted with mini prep kit (27104, Qiagen).

### ChIP

ChIP experiments were performed with a kit from Millipore (17-295, Millipore,) following the kit manual. Anti-SMAD4 antibody (sc-7154 X, Santa Cruz Biotechnology) was used to pull down SMAD4 protein, and IgG (ab37415, Abcam) served as control. Q-PCR was performed for the enrichment of SMAD4 at the promoter region of miR-503. Fold change of enrichment was calculated against input and IgG control. GAPDH promoter served as negative control. Primers are listed in Table S3.

### Collagen I contraction assay

MSCs cultured in medium (αMEM with 1% FBS) with or without 5 ng/ml TGFβ1 were detached and then mixed with collagen I solution. After detachment, cells were resuspended in medium (5× αMEM with 5% FBS) at a concentration of 2 × 10^5^/ml. 100 μl of the cell suspension was later mixed with 200 μl of empty αMEM and 200 μl of collagen I reagent (3440-100-01, R&D Systems). The mixture was later neutralized with 5 μl of 1 m NaOH. The final concentration of the collagen I was 2 mg/ml. 500 μl of the final mixture was placed in 24-well plates for 24 h before the gel was formed and then detached with the pipette tips. Pictures were taken 5 h after the gel detachment. The surface area of the gel was measured with ImageJ.

### Subcutaneous Matrigel plug assay

0.5 million MSC-derived SMCs were mixed with 0.5 million human umbilical vein endothelial cells in 100 μl of Matrigel and injected subcutaneously into SCID mice. The same number of undifferentiated cells mixed with umbilical vein endothelial cells were used as control. Matrigel plugs were harvested 2 weeks after the injection and subjected to cryostat sectioning. H&E staining and immunofluorescent staining were carried out to examine the vasculogenesis capacity of the cell mixture. Four Matrigel plugs were obtained in each group.

### Cell seeding and vascular graft engineering with the bioreactor system

Cell seeding and bioreactor establishment were conducted following the previously established protocol with minor modifications (28). Briefly, differentiated MSCs were seeded on aortic scaffold that was previously decellularized with 0.075% SDS. The scaffold was preconditioned in culture media, and then 1.5 million of differentiated MSCs were resuspended in 100 μl of Matrigel. Resuspended cells were seeded onto the scaffold, and the chamber was maintained at room temperature until the Matrigel was solidified. Shear stress was applied to the bioreactor by applying 3 ml/min fluid speed to the system. After 1 week, the graft was harvested and cryo-sectioned for H&E and immunofluorescent staining.

### Statistical analysis

Data were shown as the mean ± S.D. generated by GraphPad Prism 6 software (GraphPad Software Inc.). Data between the two groups with normal distribution were analyzed with unpaired and ungrouped *t* test, and data across multiple groups were analyzed with one-way ANOVA test, followed by Bonferroni post hoc analysis. *p* value <0.05 was considered statistically significant.

### Study approval

All animal procedures were performed according to protocols approved by the Institutional Committee for Use and Care of Laboratory Animals, and the license was issued by Home Office UK. Guidelines from Directive 2010/63/EU of the European Parliament on the protection of animals were followed.

## Author contributions

W. G., X. H., A. L. B., W. N. N., J. D., Y. X., Y. H., and Q. X. conceptualization; W. G. resources; W. G., X. H., and W. N. N. data curation; W. G. software; W. G. formal analysis; W. G., X. H., A. L. B., S. I. B., and J. D. methodology; W. G. writing-original draft; W. G. and Q. X. project administration; W. G., X. H., A. L. B., W. N. N., S. I. B., J. D., Y. X., Y. H., X. Z. R., and Q. X. writing-review and editing; X. H., A. L. B., and Q. X. supervision; S. I. B. and Q. X. validation; Y. H. and X. Z. R. funding acquisition.

## Supplementary Material

Supporting Information

## Footnotes

5 Please note that the JBC is not responsible for the long-term archiving and maintenance of this site or any other third party hosted site.

## Footnotes

4 The abbreviations used are:

SMC: smooth muscle cell
MSC: mesenchymal stem cell
HUVEC: human umbilical cord endothelial cells
miR-: microRNA
TGF-β1: transforming growth factor-β 1
ROCK: Rho-associated protein kinase
αSMA: smooth muscle α-actin
SMMHC: smooth muscle myosin heavy chain
α-MEM: α-minimal essential medium
BisTris: 2-[bis(2-hydroxyethyl)amino]-2-(hydroxymethyl)propane-1,3-diol
DAPI: 4′,6-diamidino-2-phenylindole
SCID: severe combined immunodeficient
FBS: fetal bovine serum
FGF: fibroblast growth factor
rh: recombinant human
Q-PCR: quantitative PCR
ANOVA: analysis of variance
SCID: severe combined immunodeficient.
