# Supplementary material for: Smooth muscle cells differentiated from mesenchymal stem cells are regulated by microRNAs and suitable for vascular tissue grafts
Source: J Biol Chem. 2018 Apr 11;293(21):8089–102. doi: 10.1074/jbc.RA118.001739 (PMC5971462; doi:10.1074/jbc.RA118.001739)
Supplement: Supporting Information [file supp_293_21_8089__index.html]

Smooth muscle cells differentiated from human mesenchymal stem cells regulated by microRNA (miR)-503 and miR-222-5p are suitable for vascular tissue engineering — miRNA and smooth muscle differentiation — Smooth muscle cells differentiated from mesenchymal stem cells are regulated by microRNAs and suitable for vascular tissue grafts — miRNA and smooth muscle differentiation — Supporting Information 

# Smooth muscle cells differentiated from mesenchymal stem cells are regulated by microRNAs and suitable for vascular tissue grafts

## Supporting Information

- Smooth muscle differentiation via miRNA-related pathways - Supporting tables and figures
