# Supplementary material for: Smooth muscle cells differentiated from mesenchymal stem cells are regulated by microRNAs and suitable for vascular tissue grafts
Source: J Biol Chem. 2018 Apr 11;293(21):8089–102. doi: 10.1074/jbc.RA118.001739 (PMC5971462; doi:10.1074/jbc.RA118.001739)
Supplement: Supporting Information [file supp_RA118.001739_135056_2_supp_110967_p6mxkx.pdf]

Table S1. Primer sequence used in Q-PCR

Gene Symbol	Sequence (5'→3')	Ct value range
**Human GAPDH**	F: 5-CAT GTT CGT CAT GGG TGT GAA CCA-3<br>R: 5-ATG GCA TGG ACT GTG GTC ATG AGT-3	17-19
**Human calponin**	F: 5-TTG AGG CCA ACG ACC TGT TTG AGA-3<br>R: 5-TCG AAT TTC CGC TCC TGC TTC TCT-3	20-25
**Human SM22**	F: 5-TTG AAG GCA AAG ACA TGG CAG CAG-3<br>R: 5-TCC ACG GTA GTG CCC ATC ATT CTT-3	18-22
**Human αSMA**	F: 5-TGA CAA TGG CTC TGG GCT CTG TAA-3<br>R: 5-TTC GTC ACC CAC GTA GCT GTC TTT-3	20-23
**Human Collagen I**	F: 5-CTG CAA AGG CAG CCA AAT AC-3<br>R: 5-ACA CCA AAG CCG GGA AA-3	29-32
**Human SRF**	F: 5-TGA GTG CCA CTG GCT TTG AAG AGA-3<br>R: 5-AGA GGT GCT AGG TGC TGT TTG GAT-3	20-23
**Human Myocardin**	F: 5-TTG AAA GCG GAG AAA TGC CAG CAG-3<br>R: 5-ACT GTC GGT GGC ATA GGG ATC AAA-3	20-23
**Human SMMHC**	F: 5-ATC CAT CCT CAC TCC TCG TAT C-3<br>R: 5-CCA AAG CCT CTA CAG CAA AGT-3	27-31
**Human Elastin**	F: 5-TGT TCC TGG ACT TGG AGT TG-3<br>R: 5- GCT CCA TAT TTG GCT GCT TTA G-3	29-32

Table S2. Mutation primer of the plasmids containing 3'-UTR segments of specific gene

3'-UTR	Mutation primer
**hSmad7 m1**	1: 5'-AGCACTCAGGAGGAAAATATTACGTGCAAAGTAGTTTGAAGTGTGGC-3'<br>2: 5'-GCCACACTTCAAACTACTTTGCACGTAATATTTTCCTCCTGAGTGCT-3'
**hSmad7 m2**	1: 5'-GCAAGCACTCAGGAGGAAAATATGATCTCCAAAGTAGTTTGAAGTGTGGCCT-3'<br>2: 5'-AGGCCACACTTCAAACTACTTTGGAGATCATATTTTCCTCCTGAGTGCTTGC-3'
**hROCK 2 m1**	1: 5'-CAAAACCAGTTTCAGTCTATCTGCACGCCAGTAGCTACTCTTCAGTT-3'<br>2: 5'-AACTGAAGAGTAGCTACTGGCGTGCAGATAGACTGAAACTGGTTTTG-3'
**hROCK 2 m2**	1: 5'-GGGAAGGAGAGGTGAGTCTGCACCAGTGTCAATGCAGACTC-3'<br>2: 5'-GAGTCTGCATTGACACTGGTGCAGACTCACCTCTCCTTCCC-3'
**hαSMA m**	1: 5'-CTGCTCGAACTAGTCTCGAGACCACTCCCCTATTTCAGATTTATTAAAAAAC-3'<br>2: 5'-GTTTTTTAATAAATCTGAAATAGGGGAGTGGTCTCGAGACTAGTTCGAGCAG-3'

Table S3. Primers for GAPDH and miR-503 promoter

Gene promoter	primer
**GAPDH**	F: 5'-GGC CTC CAA GGA GTA AGA CC-3'<br>R: 5'-AGG GGT CTA CAT GGC AAC TG-3'
**miR-503 primer 1**	F: 5'-GAG AGA AGG TAC ATC GTG TGT T-3'<br>R: 5'-CAC TTC AGG AGA GGG TCA TTC-3'
**miR-503 primer 2**	F: 5'-AAA CAG GAA GGA GCG ACT TG-3'<br>R: 5'-TCT TAC ACT ATC GTT GCG ACA TAT AC-3'
**miR-503 primer 3**	F: 5'-GAG TGA AGT GGC CTA GTC ATA AG-3'<br>R: 5'-CCT GGT GGC AGG AAC AC-3'

Figure S1

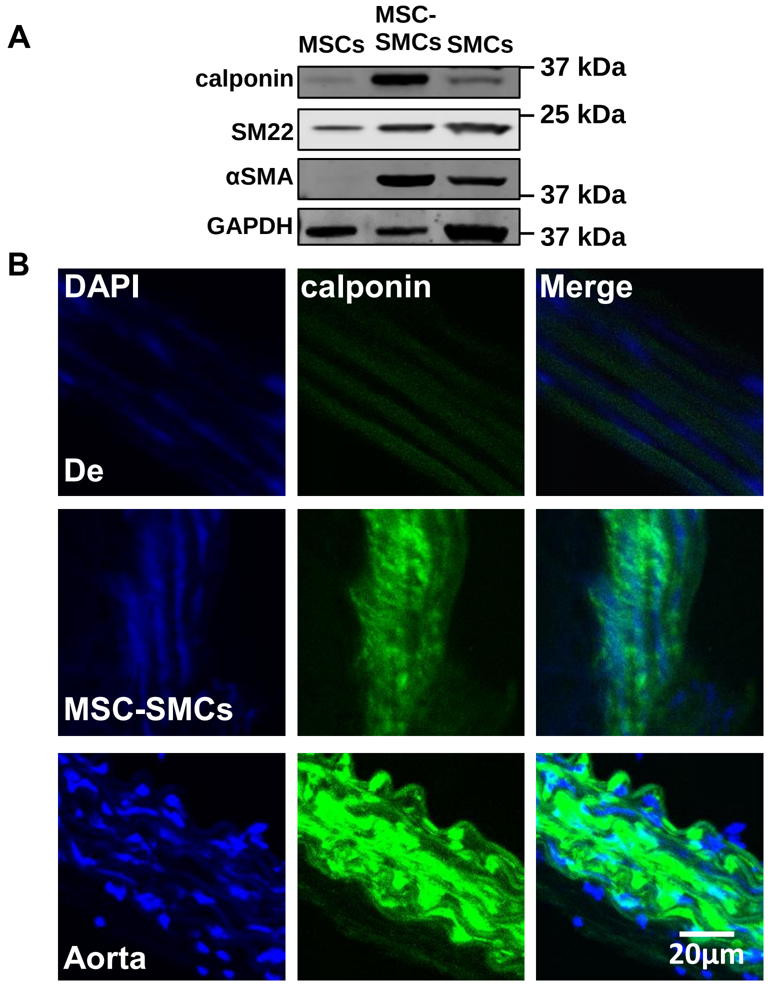

Figure S1. Differentiated SMCs from MSCs (MSC-SMCs) show comparable SMC marker level *in vitro and ex vivo*. (A) HUCMSCs were differentiated in differentiation medium (αMEM with 1% FBS and 5 ng/ml TGFβ1) for 5 days and then harvested. In comparison with *in vitro* cultured human aorta-derived SMCs, the MSC-SMCs showed strong expression of SMC markers (calponin, SM22 and αSMA) which is comparable or stronger than the cultured human SMCs. Representative image was shown from two independent experiments. (B) Comparison of DAPI and calponin staining of decellularized aorta (De) and normal aorta (Aorta) demonstrated the efficiency of decellularization. Differentiated MSCs (MSC-SMCs) were seeded onto the decellularized aorta in the bioreactor system and maintained for 5 days before harvesting for cryostat sectioning. Immunofluorescent staining of DAPI showed that the cells could recellularize to the graft in comparison with decellularized aorta. Furthermore, the graft seeded with MSC-SMCs demonstrated comparable level of calponin staining with normal aorta. Shown images are representative of two independent experimemnts.

Figure S2

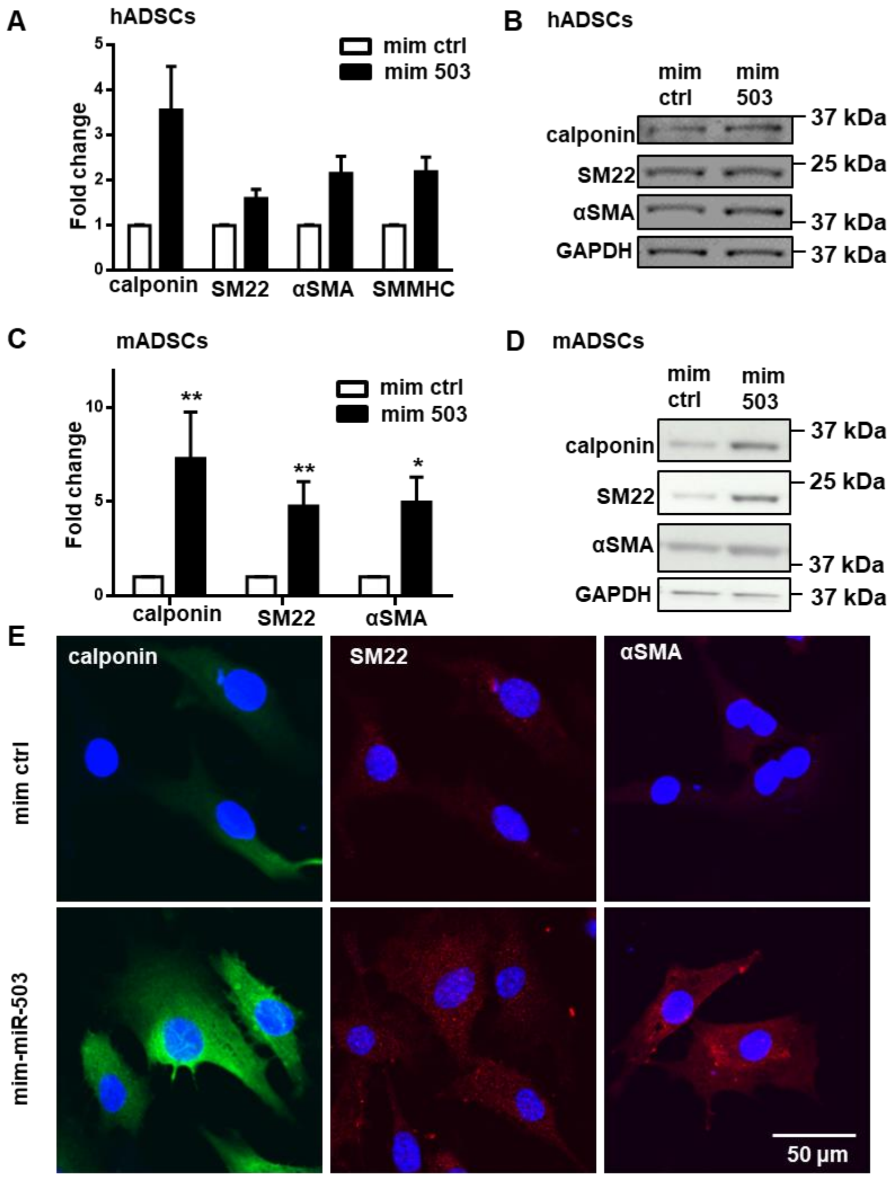

Figure S2. miR-503 promotes SMC differentiation from hADSCs and mADSCs. (A and B) hADSCs were transfected with miR-503 mimics after being seeded in medium with 1% FBS for 24 hours. Treatment lasted for 48 hours in medium with 1% FBS before the cells were harvested for Q-PCR and Western blot analysis for SMC markers. (A) Q-PCR showed the upregulation of SMC markers at the gene expression level. Data were from two independent experiments. (B) Western blot displayed the upregulation mainly for calponin and αSMA. Image shown was representative of two independent experiments. (C-E) Mouse ADSCs isolated from mouse subcutaneous adipose tissue were transfected with miR-503 mimics one day after they were seeded in αMEM with 10% FBS. The cells were further treated for 2 days before they were harvested for Q-PCR, Western blot analysis and Immunofluorescent staining. (C) Q-PCR showed the upregulation of SMC markers with miR-503 mimic treatment. Data were presented as mean ± SD and were from three independent experiments. (D) Western blot analysis demonstrated the upregulation of SMC markers at the protein level after miR-503 treatment for 2 days. Image shown was representative of three independent experiments. (E) Immunofluorescent staining showed the upregulation of SMC markers at the protein level. Images shown were representative of three independent experiments. *$p < 0.05$ and **$p < 0.01$. mim ctrl, miRNA mimic negative control; mim 503, miR-503 mimic. hADSCs, human adipose tissue-derived mesenchymal stem cells; mADSCs, mouse adipose tissue-derived mesenchymal stem cell.

Figure S3

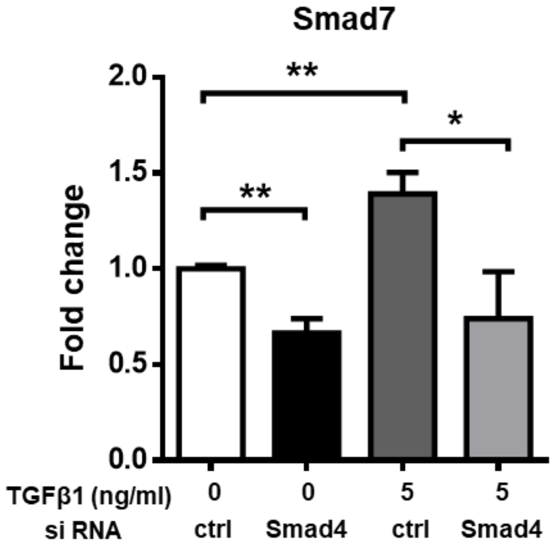

Figure S3. Smad7 is upregulated in a Smad4-dependent pathway. HUCMSCs were cultured at the density of $1x10^4/cm^2$ for 1 day in medium (αMEM with 1% FBS) before they were transfected with Smad4 siRNA. The final concentration of siRNA was 12.5 nM. Cells transfected with siRNA with or without TGFβ1 were harvested 2 days after transfection. Smad7 at the gene expression level was examined by Q-PCR. Data are obtained from at least three independent experiments and shown as mean ± SD. Statistics are obtained from one-way ANOVA followed by Bonferroni post-hoc analysis. $*p < 0.05$ and $**p < 0.01$.

Figure S4

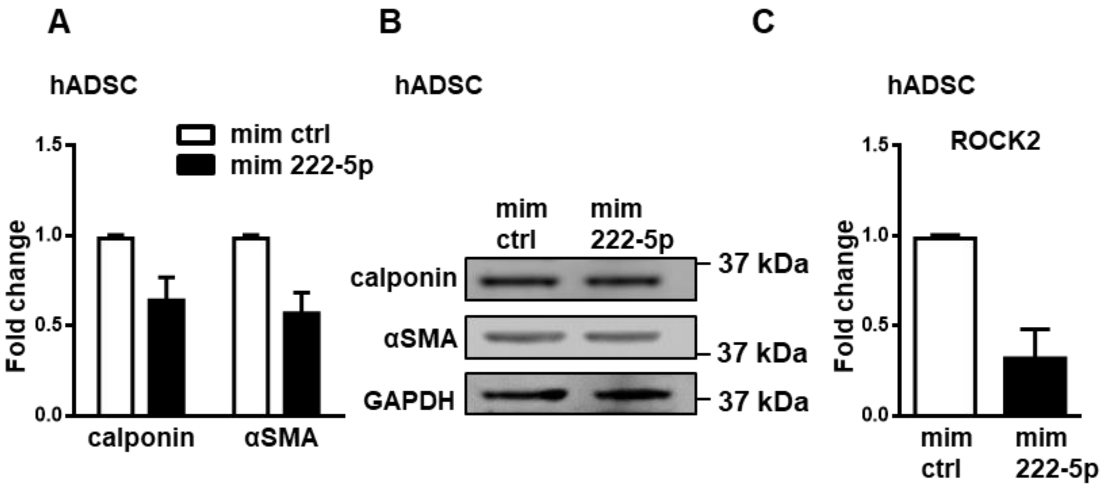

Figure S4. miR-222-5p inhibits SMC differentiation from hADSCs. hADSCs were transfected with miR-222-5p mimics after being seeded in medium with 1% FBS for 24 hours. Treatment lasted for 48 hours in medium with 1% FBS before the cells were harvested for Q-PCR and Western blot analysis. (A) Q-PCR showed the inhibition of SMC markers (calponin and αSMA) at the gene expression level. Data were shown as mean ± SD and were from two independent experiments. (B) Western blot displayed the moderate downregulation mainly for αSMA. Image shown was representative of two independent experiments. (C) Q-PCR showed the inhibition of ROCK2 with miR-222-5p mimic treatment one day after miR-222-5p mimic transfection. Data were presented as mean ± SD and were from two independent experiments. mim ctrl, miRNA mimic negative control; mim 222-5p, miR-222-5p mimic. hADSCs, human adipose tissue-derived mesenchymal stem cells.

Figure S5

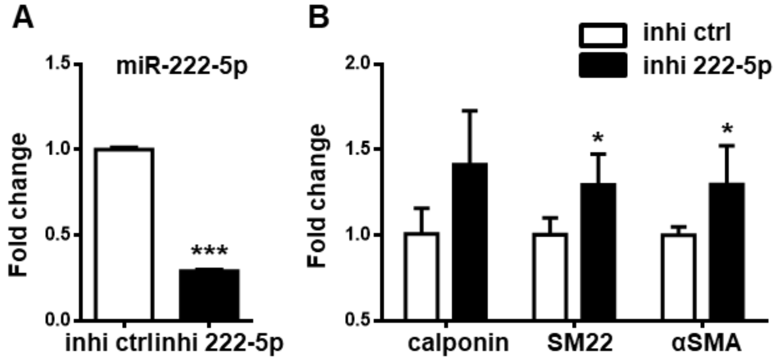

Figure S5. miR-222-5p inhibitor promotes SMC differentiation. HUCMSCs were cultured at the density of 1×10^4 /cm^2 for 1 day in medium (αMEM with 1% FBS) before they were transfected with miR-222-5p inhibitors. The final concentration of the inhibitors was 62.5 nM. Cells were harvested 2 days after transfection. Cells cultured in the same condition but transfected with miRNA inhibitor negative control was used as a control. TGFβ1 was not added to the medium in the entire process. (A) Successful inhibition of miR-222-5p level was confirmed by the significant downregulation of miR-222-5p level inside the cells. (B) It was shown that miR-222-5p inhibitor could to some extent promote SMC differentiation as demonstrated by the upregulation of SMC markers calponin, SM22 and αSMA, but the promotion ability is limited since the fold change of the SMC markers are minimal and are less than 1.5. Data are obtained from at least three independent experiments and shown as mean ± SD. Statistics are obtained from unpaired student t-test. *$p < 0.05$ and ***$p < 0.001$.
